# Sphingosine Kinase-1 (SphK-1) Regulates *Mycobacterium smegmatis* Infection in Macrophages

**DOI:** 10.1371/journal.pone.0010657

**Published:** 2010-05-17

**Authors:** Hridayesh Prakash, Anja Lüth, Natalia Grinkina, Daniela Holzer, Raj Wadgaonkar, Alexis Perez Gonzalez, Elsa Anes, Burkhard Kleuser

**Affiliations:** 1 Cell Biology and Biophysics Unit, European Molecular Biology Laboratory, Heidelberg, Germany; 2 Faculty of Mathematics and Natural Science, University of Potsdam, Berlin, Germany; 3 Centro de Patogénese, Molecular-URIA/Instituto de Medicina Molecular, Faculdade de Farmácia da, Universidade de Lisboa, Lisbonne, Portugal; 4 Flow Cytometry Core Facility, European Molecular Biology Laboratory, Heidelberg, Germany; 5 Department of Medicine, State University of New York, Brooklyn, New York, United States of America; Institut de Pharmacologie et de Biologie Structurale, France

## Abstract

Sphingosine kinase-1 is known to mediate *Mycobacterium smegmatis* induced inflammatory responses in macrophages, but its role in controlling infection has not been reported to date. We aimed to unravel the significance of SphK-1 in controlling *M. smegmatis* infection in RAW 264.7 macrophages. Our results demonstrated for the first time that selective inhibition of SphK-1 by either *D, L threo dihydrosphingosine* (DHS; a competitive inhibitor of Sphk-1) or Sphk-1 siRNA rendered RAW macrophages sensitive to *M. smegmatis* infection. This was due to the reduction in the expression of iNOs, p38, pp-38, late phagosomal marker, LAMP-2 and stabilization of the RelA (pp-65) subunit of NF-κB. This led to a reduction in the generation of NO and secretion of TNF-α in infected macrophages. Congruently, overexpression of SphK-1 conferred resistance in macrophages to infection which was due to enhancement in the generation of NO and expression of iNOs, pp38 and LAMP-2. In addition, our results also unraveled a novel regulation of p38MAPK by SphK-1 during *M. smegmatis* infection and generation of NO in macrophages. Enhanced NO generation and expression of iNOs in SphK-1++ infected macrophages demonstrated their M-1^bright^ phenotype of these macrophages. These findings thus suggested a novel antimycobacterial role of SphK-1 in macrophages.

## Introduction

Sphingolipids have recently been identified as crucial bioactive molecules in several fundamental and patho-physiological processes [1], [2]. A novel therapeutic potential of sphingolipids has been documented for the treatment of asthma, cystic fibrosis, respiratory tract infection and acute lung injuries [3]–[6]. Sphingolipids are known to regulate cellular functions differentially. Thus, while sphingosine 1-phosphate (S1P) promotes cell survival and cell division [7], ceramides and sphingosine inhibit them and induce apoptosis [2], [8]. The sphingolipids are interconvertible, suggesting that sphingolipid metabolism is closely regulated. Sphingosine kinases (SphKs), which catalyze the phosphorylation of sphingosine to S1P, are enzymes crucial to sphingolipid metabolism [9]. Two subtypes of SphKs have been identified to date, namely SphK-1 and SphK-2 [10]. Among these, SphK-1 is a well known regulator of intracellular calcium homeostasis, cellular differentiation, innate immunity, apoptosis and cancer development [8], [11]–[17], while the role of SphK-2 remains unclear.

Recent reports have demonstrated the involvement of SphK-1 during mycobacterial infections in macrophages [18]–[20]. During the course of infection, the mycobacteria containing phagosomes are processed and mature to phagolysosomes. These organelles are rich in hydrolytic enzymes and anti-mycobacterial mediators which execute the killing of these mycobacteria in macrophages [21]–[24]. It has been demonstrated that during mycobacterial infection, SphK-1 translocates to the phagosomal membrane where it creates a pro-inflammatory environment mainly by inducing actin nucleation [25]–[27]. This is a prerequisite for the efficient killing by a variety of macrophages of both non-pathogenic and pathogenic mycobacteria [28]–[30] as demonstrated recently by our former co-workers [23], [24]. *Mycobacterium smegmatis* infections activate resting macrophages to pro-inflammatory and antibacterial M-1 macrophages [31]. Among various mediators which are secreted by these macrophages, TNF-α and inducible NO are critical for limiting mycobacterial infections [32]–[34]. These are known to induce maturation of mycobacteria containing phagolysosomes and intracellular killing of these bacteria in macrophages [35], [36].

Although the involvement of SphK-1 during *M. smegmatis* infection in macrophages has been previously demonstrated [18], its direct role in controlling infection has not been reported so far. This study therefore demonstrates for the first time that inhibition of SphK-1 rendered RAW macrophages sensitive to infection. This was due to the reduced expression of major anti-mycobacterial proteins such as iNOs, p38, pp38 and late phagolysosomal marker, LAMP-2 and reduced activation of NF-kB in the infected macrophages. In addition, the generation of NO and TNF-α secretion were also reduced upon Sphk-1 inhibition in infected macrophages. Conversely and expectedly, SphK-1 overexpression conferred resistance to infection and enhanced expression of iNOS, pp38 and LAMP-2 proteins in Sphk-1++ macrophages. Sphk-1 overexpression also led to an enhancement in the generation of NO, but interestingly delayed secretion of TNF-α. Our data also demonstrated the novel regulation of SphK-1 over p38 for controlling infection and the generation of NO in macrophages. Enhanced generation of NO and increased expression of iNOs protein in SphK-1++ macrophages in response to *M. smegmatis* and/or various innate stimuli demonstrated their M-1^bright^ phenotype. These findings thus suggest a new antimycobacterial and immunostimulatory role of SphK-1 in macrophages.

## Results

### SphK-1 controls M. smegmatis infection in macrophages

**Figure 1:**
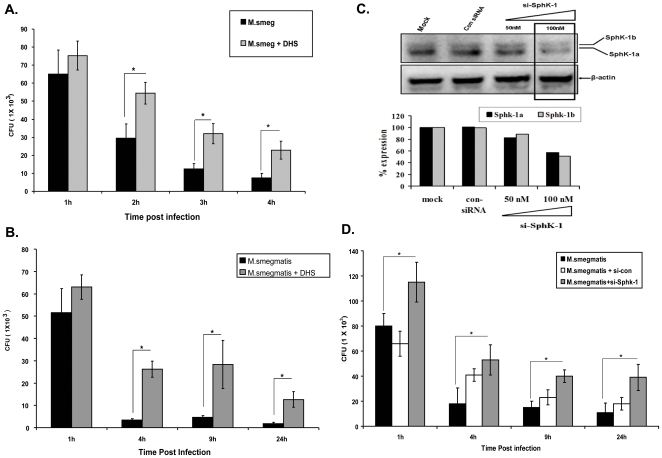
Sensitization of SphK-1 inhibited RAW macrophage to *M. smegmatis* infection. The macrophages were treated with DHS and the effect of DHS treatment on *M. smegmatis* killing was evaluated up to 4 h (**A**) and 24 h (**B**) post infection. (**C**) siRNA knockdown of Sphk-1 in macrophages. The macrophages were transfected with SphK-1 specific and control, siRNA's and knockdown was confirmed by western blot 24 h post transfection. Shown here is the representative blot from two independent experiments. (**D**) Sensitization of Sphk-1 knockdown macrophages to *M. smegmatis* infection. Control, scrambled siRNA and Sphk-1 siRNA transfected macrophages were infected with *M. smegmatis* at MOI-1 and their killing was monitored up to 24 h post infection. Data are represented as a mean of CFU ± SEM from three independent experiments. ** Indicates p≤0.01; * indicates p≤0.05.

A recent report has shown the involvement of SphK-1 during *M. smegmatis* induced pro-inflammatory responses in macrophages [18]. Based on this information we presumed that Sphk-1 is an important component of macrophage defense against infection and predicted that Sphk-1 could also control *M. smegmatis* infection. To demonstrate this, we used an *M. smegmatis* infected RAW 264.7 murine macrophage model system. These macrophages were infected with *M. smegmatis* and bacterial killing was monitored over a time period of 24 h. Indeed, we observed efficient intracellular bacterial killing during the first 4 h of infection (**Fig. 1A** **, Fig. S1**). In order to demonstrate the specific role of SphK-1 in controlling *M. smegmatis* infection, we inhibited SphK-1 in macrophages by ***D, L-threo-dihydrosphingosine (DHS)*** [37]–[39], and ***Sphk-1 siRNA***. These macrophages were then infected with *M. smegmatis*. Indeed, inhibition of Sphk-1 either by DHS (**Fig. 1A, B**) or by siRNA (**Fig. 1D**) inhibited bacterial killing significantly and sensitized these macrophages to infection. Both macrophage viability and metabolic activity remained unaffected by the doses of DHS used (**Fig. S2**) and siRNA (data not shown) knockdown. In order to demonstrate the specific effect of Sphk-1 inhibition on bacterial killing, the macrophages were infected in the presence of other sphingosine derivatives which have no SK inhibitory activity, e.g. ***D- sphingosine*** **and** ***D, L-erythro–dihydrosphingosine***. Bacterial killing remained unaffected by the treatment of macrophages with these lipids (**Fig. S3**).

**Figure 2:**
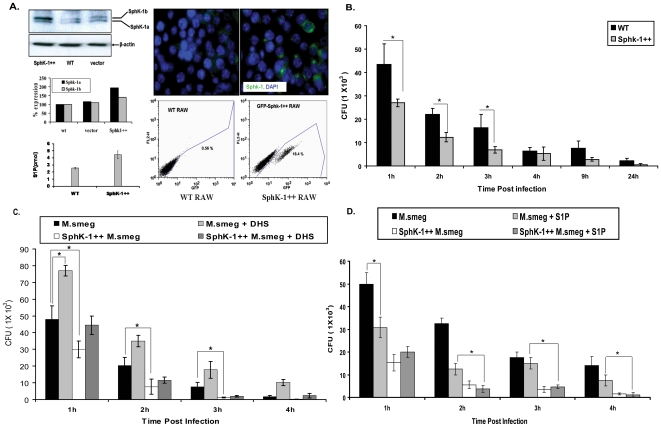
SphK-1 overexpression confers resistance to *M. smegmatis* infection in macrophages. (**A**) Sphk-1 was overexpressed in macrophages and validated by western blot, immunofluorescence and competitive S1P titers in WT and SphK-1++ macrophages. (**B**) Sphk-1 overexpression confers resistance to infection. Both WT and SphK-1 ++macrophages were infected with *M. smegmatis* and mycobacterial killing was observed up to 24 h post infection. (**C**) The cells under section (**B**) were treated with DHS and the effect on *M. smegmatis* killing was again evaluated up to 24 h post infection. (**D**) S1P regulates mycobacterial growth in macrophages. The cells under section (**B**) were supplemented with S1P (5 µM) and the effect of S1P on mycobacterial infection was monitored during the first 4 h time period. Data are represented as mean of CFU ± SEM from three independent experiments. ** Indicates p≤0.01; * indicates p≤0.05.

To further substantiate whether an upregulation of SphK-1 would counteract bacterial killing, SphK-1 was overexpressed in macrophages (**Fig. 2A**) and these macrophages (Sphk-1++) were infected with *M. smegmatis*. Interestingly and expectedly, SphK-1 overexpression conferred resistance to infection in Sphk-1++ macrophages in comparison to WT infected macrophages (**Fig. 2B**). The increased resistance of SphK-1++ infected macrophages was abolished by DHS treatment (**Fig. 2C**). Treatment of both WT and Sphk-1++ infected macrophages with S1P (SK reaction product) enhanced bacterial killing in both WT and SphK-1++ infected macrophages. This effect was found more pronounced in SphK-1++ infected macrophages than in WT infected macrophages (**Fig. 2D**). These observations confirmed the involvement of SphK-1 in controlling *M. smegmatis* infection in macrophages.

### SphK-1 regulates antimycobacterial response in macrophages

**Figure 3:**
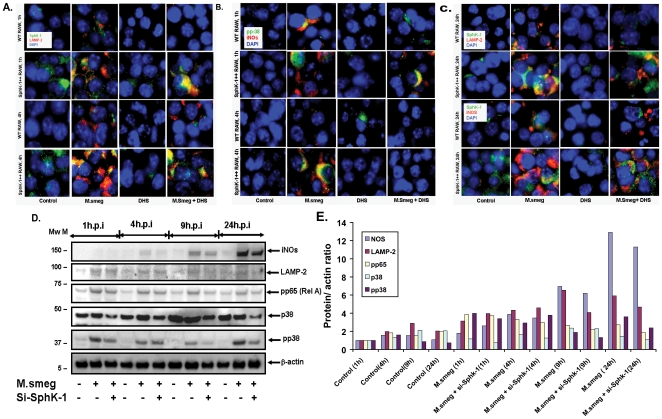
SphK-1 regulates the expression of major antimycobacterial proteins in macrophages. WT and SphK-1++ macrophages were infected with *M. smegmatis* with and without DHS. Expression of LAMP-2 protein was monitored at 1 h and 4 h (**A**) and 24 h (**C**) post infection. The cells under sections (**A**) and (**C**) were also analyzed for the expression of pp38 at 1 h and 4 h (**A**) and iNOs at 1 h, 4 h (**B**) and 24 h (**C**) post infection time intervals. Shown here are the representative immunofluorescence pictures from two independent experiments. (**D**) Sphk-1 knockdown reduces the expression of major antimycobacterial proteins in macrophages. Both control and Sphk-1 siRNA knockdown macrophages were infected with *M. smegmatis* and expression of major antimycobacterial proteins was analyzed by western blot. Shown here is the representative blot from two independent experiments. (**E**) The blots were normalized against actin.

Sensitization of Sphk-1 depleted macrophages and increased resistance of Sphk-1++ macrophages to *M. smegmatis* infection clearly indicated the involvement of SphK-1 in the antimycobacterial defense of macrophages. To address this, we analyzed the expression of the late phagolysosomal marker LAMP-2 (**Fig. 3A**) and anti-mycobacterial proteins- pp38 and iNOs (**Fig. 3B, C**) competitively among WT and SphK-1++ infected macrophages. Immunostaining revealed an increase in the expression of these markers in SphK-1++ infected macrophages in comparison to WT infected macrophages during the course of infection. Indeed, Sphk-1 inhibition by DHS reduced expression of these proteins in infected macrophages (**Fig. 3A, B and C**). Remarkably, siRNA knockdown of Sphk-1 also reduced *M. smegmatis* induced expression of iNOs, p38, p-p38, LAMP-2 and pp65 (Rel A) subunit of NF-κB (**Fig. 3D**) in comparison to mock (scrambled) transfected or untransfected macrophages. These results revealed the influence of SphK-1 on antimycobacterial defenses of macrophages. Enhanced expression of LAMP-2, pp38 and iNOs proteins in SphK-1 ++ infected macrophages over WT infected macrophages demonstrated the increased antimycobacterial response of Sphk-1++ macrophages, which rendered them resistant to the infection.

### SphK-1 is involved in nitric oxide generation in macrophages

**Figure 4:**
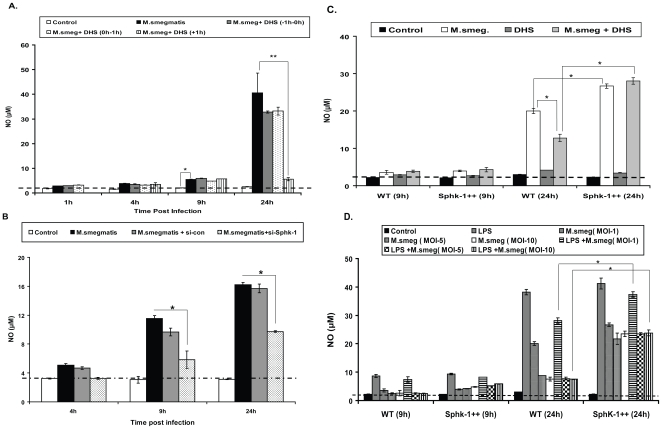
SphK-1 regulates *M. smegmatis* infection induced NO generation in macrophages. (**A**) WT macrophages were infected with *M. smegmatis* with and without DHS for indicated time intervals and their culture supernatants were collected to measure inducible NO as NO_2_ as described. (**B**) Sphk-1 knockdown inhibits *M. smegmatis* induced NO generation. Both control and Sphk-1 siRNA knockdown macrophages were infected with *M. smegmatis* and NO was quantified in their culture supernatant at indicated time intervals. (**C**) Complementation of NO by SphK-1 overexpression in macrophages. Both WT and SphK-1++ macrophages were infected with *M. smegmatis* with and without DHS and NO was quantified in the culture supernatants at indicated time points. (**D**) SphK-1 overexpression restores the functional integrity of NO response in infected macrophages. Both WT and SphK-1++ macrophages were infected with increasing doses of *M. smegmatis* and co-stimulated with LPS (positive control). The culture supernatants were collected at indicated time intervals and NO was quantified. The dotted line in all figures represents the constitutive NO titre in macrophages. The values above this line represent the actual titre of NO being induced by various treatments. ** Indicates p≤0.01; * indicates p≤0.05.

In order to clarify the potential reason for sensitization of Sphk-1 inhibited macrophages to infection, we investigated the effect of SphK-1 inhibition on *M. smegmatis* infection induced NO generation in macrophages due to the fact that NO is a well known marker for classically activated M-1 professional macrophages and is involved in antibacterial defenses [40]–[45]. In macrophages, NO is produced by the iNOs enzyme and capable of neutralizing a wide variety of mycobacterial membrane lipids and other components and indeed is involved in maturation of phagosomes during mycobacterial infection [46]–[48]. During the first 4 h time period of infection, no significant increase in NO was observed in the infected macrophages. The NO titre increased slightly at the 9^th^ h post infection and significantly at the 24^th^ h post infection in comparison to the uninfected control (**Fig. 4A**). Inhibition of SphK-1 significantly reduced *M. smegmatis* induced NO titre during the course of infection (**Fig. 4A**). siRNA knockdown of Sphk-1 also reduced infection induced NO titre significantly in comparison to mock (scrambled) transfected or untransfected control (**Fig. 4B**). Interestingly, Sphk-1++ macrophages, in comparison to WT macrophages, produced significantly higher NO in response to infection (**Fig. 4C**). Moreover, SphK-1 overexpression replenished DHS mediated loss of NO in macrophages (**Fig. 4C**). As expected, control sphingolipids could not modulate infection induced NO titre in macrophages (**Fig. S3C**).

The above results prompted us to investigate the role of Sphk-1 on the functional competence of these infected macrophages. To demonstrate this, we infected WT and Sphk-1++ macrophages with different multiplicities of *M. smegmatis* infection and quantified the NO titre. Interestingly, *M. smegmatis* infection inhibited NO generation in WT macrophages in a dose-dependent manner (**Fig. 4D**). In contrast, SphK-1++ macrophages were found to be resistant to such toxicity of infection and the NO titre in Sphk-1++ infected macrophages remained significantly higher than WT infected macrophages (**Fig. 4D**). *M. smegmatis* interacts with macrophages and activates them through TLR2 and MyD88 adaptor proteins [49]. Our NO data suggested a possible association of SphK-1 with these innate receptors for activating macrophage. In order to validate this, we stimulated these macrophages with unrelated bacterial LPS (positive control) [50]–[52] and NO was measured. The NO titre in Sphk-1++ infected and LPS stimulated macrophages consistently remained higher in comparison to WT macrophages (**Fig. 4D**). These observations explicitly demonstrated that SphK-1 is critical for NO response and sustained activation of macrophages even under conditions of supra-lethal infection.

**Figure 5:**
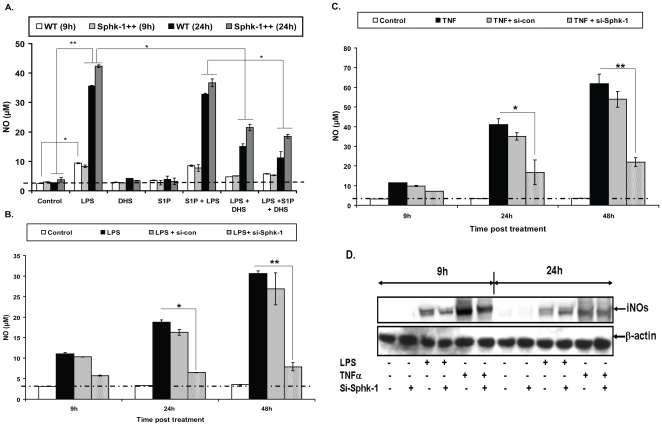
Sphk-1 are also involved in LPS/TNF induced NO generation in macrophages. (**A**) WT and SphK-1++ macrophages were stimulated with LPS with and without S1P/DHS and NO was quantified at indicated time intervals. (**B, C**) Sphk-1 knockdown inhibits LPS and TNF-α induced generation of NO in macrophages. Both control and Sphk-1 knockdown macrophages were stimulated with LPS (**B**) and TNF-α (**C**) and NO was quantified at indicated time intervals. (**D**) The effect of Sphk-1 knockdown was validated in LPS or TNF-α induced generation of NO and expression of iNOs proteins at different time intervals. Shown here is the representative blot from two independent experiments. Data in all figures are represented as mean of µM ± SEM from three independent experiments. The dotted line in all figures represents the constitutive NO titre in macrophages. The values above this line represent the actual titre of NO induced by various treatments. **Indicates p≤0.01; * indicates p≤0.05).

**Figure 6:**
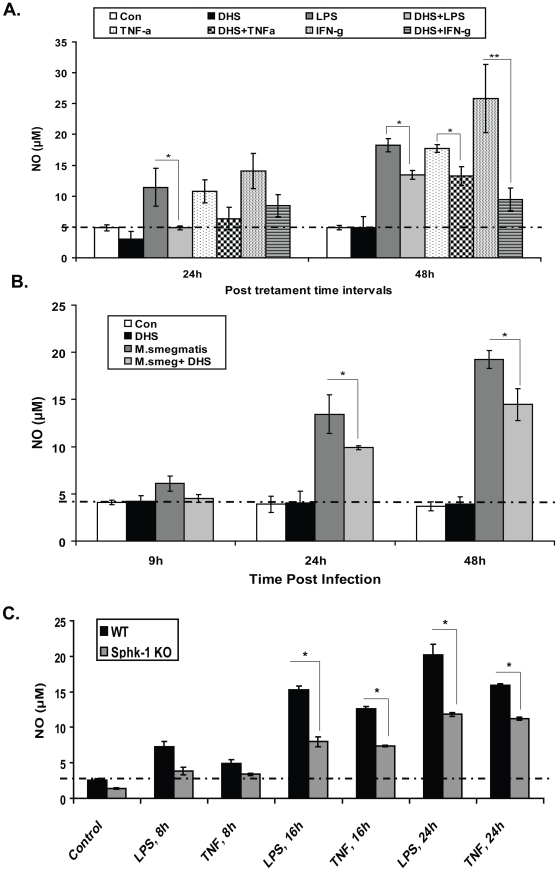
Verification of the role of Sphk-1 on the generation of NO in primary mouse macrophages. (**A**) Mac-1+ peritoneal macrophages were isolated from WT mice and stimulated with LPS/TNFα/IFN-γ with and without DHS and the NO was quantified at 24 and 48 h post treatment. (**B**) The Mac-1+ peritoneal macrophages were infected with *M. smegmatis* as described both in the presence or absence of DHS and NO was quantified at indicated post infection time intervals. (**C**) Mac-1+ peritoneal macrophages were isolated from both WT and Sphk-1 KO mice and stimulated with both LPS and TNF-α. NO was quantified in their culture supernatant at indicated time intervals. Data in all figures are represented as mean of µM ± SEM from three independent experiments. The dotted line in all figures represents the constitutive NO titre in macrophages. The values above this line represent the actual titre of NO induced by various treatments. ** Indicates p≤0.01; * indicates p≤0.05.

To further demonstrate the involvement of SphK-1 in NO generation via innate signaling pathways, we investigated the effect of Sphk-1 inhibition on LPS induced NO generation. To show this, WT and SphK-1++ macrophages were stimulated with LPS in the presence of DHS and/or S1P and the NO titre was quantified. Treatment of LPS stimulated macrophages with DHS inhibited LPS induced NO generation significantly (**Fig. 5A**). The NO titers remained consistently higher in SphK-1++ stimulated macrophages in comparison to WT macrophages (**Fig. 5A**). Although exogenous supplementation of macrophages with S1P did not affect LPS induced NO generation in either of these macrophages (**Fig. 5A**), it inhibited *M. smegmatis* induced NO significantly in both (**Fig. S4A**). SiRNA knockdown of Sphk-1 also inhibited either LPS (**Fig. 5B**) or TNF-α (**Fig. 5C**) induced NO titre significantly as well as expression of iNOs proteins in comparison to either mock transfected (scrambled siRNA) or untransfected control macrophages **(** **Fig. 5D** **)**. Simultaneous treatment of LPS stimulated/infected and S1P supplemented macrophages with DHS again diminished NO titre in both these hosts (**Fig. S4A**). In comparison to WT macrophages, SphK-1++ macrophages produced significantly higher NO upon their stimulation with TNF-α and IFN-γ both in the presence or absence of iNOS modulators (**Fig. S4B, C**). Immunoblot analysis also revealed a downregulation in the expression of iNOs proteins by DHS in LPS/TNFα/IFN-γ stimulated macrophages (**Fig. S4D**). Control sphingolipids were unable to modulate either LPS (**Fig. S5A**) or TNF-α (**Fig. S5B**) induced NO titre in macrophages and excluded the Sphk-1 unspecific effect on NO generation. Next we additionally validated the role of Sphk-1 in NO in mouse primary macrophages. For that purpose we isolated Mac-1+ mouse peritoneal macrophages from C57BL6/j mice as per the method described. Treatment of LPS stimulated (**Fig. 6A**) or infected (**Fig. 6B**) CD11b+ mouse peritoneal macrophage with DHS reduced NO titre in these macrophages. To further confirm the novel role of SphK-1 on NO generation, we compared the NO titre among stimulated WT and SphK-1 KO Mac-1+ peritoneal macrophages. As expected, Sphk-1 KO peritoneal macrophages produced significantly less NO in comparison to WT macrophages upon their stimulation with either LPS or TNF-α (**Fig. 6C**). These results verified and confirmed the novel and specific regulation of SphK-1 in the generation of NO in macrophages.

### SphK-1 regulates p38 MAPK in macrophages during mycobacterial infection

**Figure 7:**
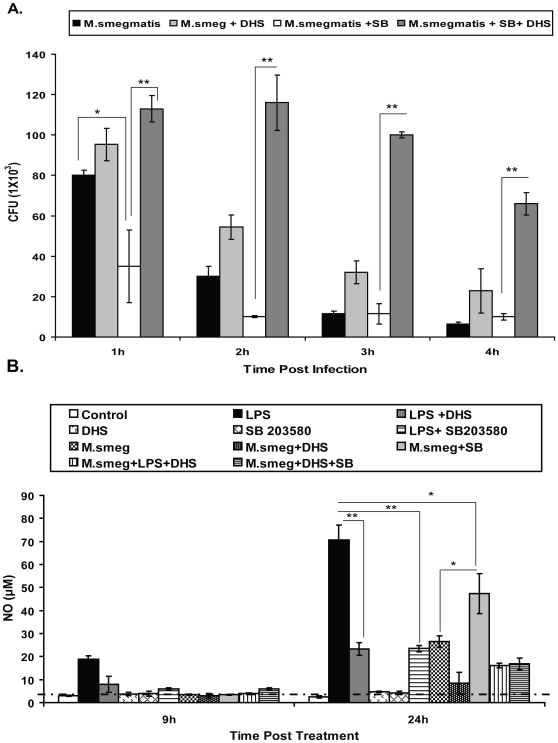
Sphk-1 regulates p38 MAPK in infected macrophages. (**A**) The macrophages were treated with p38MAPK inhibitor (SB 203580; 2 µM) with and without DHS and infected with *M. smegmatis* and mycobacterial killing was monitored over a 4 h period. Data are represented as a mean of CFU ± SEM from three independent experiments. ** Indicates p≤0.01; * indicates p≤0.05. (**B**) SphK-1 is involved in p38 and *M. smegmatis* mediated generation of NO in macrophages. The macrophages were infected with *M. smegmatis* with and without SB 203580 and DHS, either separately or together. The LPS was used as positive control and NO was quantified. Data are represented as mean µM ± SEM from three independent experiments. The dotted line represents the constitutive NO titre in macrophages. The values above this line represent the actual titre of NO induced by various treatments. ** Indicates p≤0.01; * indicates p≤0.05.

Previous studies have demonstrated the critical role of p38 in controlling *mycobacterial* infection in macrophages other than RAW [24], [53]. The p38 activation during *M. smegmatis* infection was shown to be dependent either on the cell type and/or the bacterial load [54]. Here we found that in opposition to J774 macrophage [24], p38 inhibition in RAW macrophages surprisingly reduced both bacterial uptake and viability (**Fig. 7A**). This revealed the different fate of p38 in RAW macrophages upon infection, since similar inhibition in J774 macrophages promoted intracellular growth of these bacteria. Based on the results obtained from RAW macrophages, we predicted a possible connection of SphK-1 with p38 for regulating bacterial infection. In order to investigate this, we treated an infected RAW macrophage with SB 203580 (p38kinase inhibitor) with and without DHS (Sphk-1 inhibitor) and bacterial killing was monitored. Surprisingly, co-inhibition of p38 and SphK-1 increased both bacterial uptake and their growth significantly (**Fig. 7A**) in comparison to infected macrophages treated with SB 203580 alone.

Besides regulating mycobacterial infection [53], p38 are also potent regulators of inducible NO generation [55], [56]. Our data (**Fig. 4** **,** **5** **,** **6**) clearly demonstrated the regulation of NO by Sphk-1 in macrophages. We therefore predicted the probable connection of Sphk-1 and p38 for NO generation in macrophages. In order to clarify this, the macrophages were infected with and without p38 and Sphk-1 inhibitors and NO titre was quantified. Treatment of infected macrophages with SB 203580 surprisingly enhanced infection induced NO titre significantly in comparison to infected controls (**Fig. 7B**). Treatment of SB+ infected macrophages with DHS strongly inhibited NO titre in these macrophages. These results strongly suggested that Sphk-1 regulates p38 during *M. smegmatis* induced NO generation in macrophages. These results demonstrated a novel regulation of p38 by Sphk-1 in macrophages during the course of infection.

### SphK-1 regulates TNF-α secretion in macrophages

**Figure 8:**
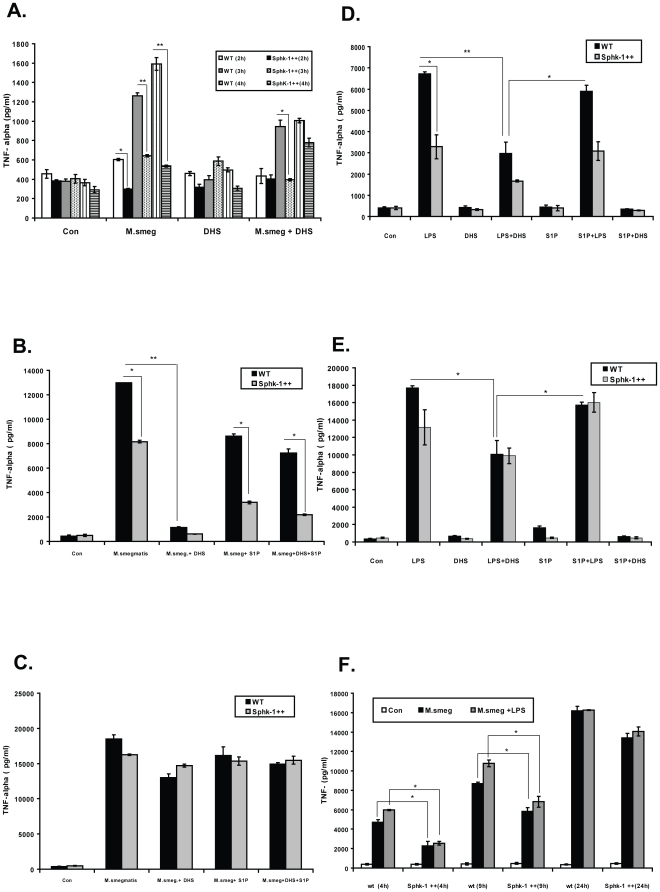
SphK-1 regulates TNF-α secretion in macrophages. (**A**) Both WT and SphK-1++ macrophages were infected with *M. smegmatis* with and without DHS. The TNF-α titre was quantified in their culture supernatants until 4 h post infection. (**B,C**) Delay in the secretion of TNF-α by S1P in macrophages. The macrophages were infected with *M. smegmatis* with and without DHS (20 µM) and S1P (5 µM), and their culture supernatants were collected at 9 h (**B**) and 24 h (**C**) post infection and TNF-α titre was quantified. (**D**) The macrophages were stimulated by LPS with and without S1P and DHS, TNF titre was quantified at 9 h (**D**) and 24 h (**E**) post treatment. (**F**) Sphk-1 overexpression delays in the secretion of TNF-α by infected macrophage upon LPS co-stimulation. Both WT and Sphk-1++ macrophages were infected with *M. smegmatis* and co-stimulated with LPS for indicated time intervals. The TNF titre was quantified. Data are represented as a mean ρg/ml ± SEM from three independent experiments. ** Indicates p≤0.01; * indicates p≤0.05.

TNF-*α* is a well known trigger of acute inflammatory response during mycobacterial infection and largely secreted by activated macrophages [57], [58]. Therefore, we investigated the effect of SphK-1 modulation on TNF-α secretion also. Treatment of infected macrophages with DHS significantly reduced *M. smegmatis* induced TNF-α secretion in WT infected macrophages in comparison to infected controls (**Fig. 8A**). This observation explained to some extent the most probable reason for the sensitization of Sphk-1inhibited macrophages to infection. On the basis of increased resistance to infection, we predicted enhanced TNF-α secretion in Sphk-1++ infected macrophages over WT infected macrophages. While we were able to detect higher TNF-α secretion in SphK-1++ infected macrophages over WT infected macrophages at 1 h post infection, this remained insignificant (data not shown). Interestingly, and contrary to our expectations, the TNF-α secretion in Sphk-1++ infected macrophages was significantly reduced in comparison to WT infected macrophages during later time points of infection (**Fig. 8A**). This observation revealed negative regulation of Sphk-1 over-expression on TNF-α secretion. In order to complement Sphk-1 inhibition mediated loss in TNF-α secretion in macrophages, we supplemented infected macrophages exogenously with S1P (SK reaction product). Surprisingly, this supplementation delayed TNF-α secretion in the infected macrophages at 9 h (**Fig. 8B**), which was restored at 24 h post infection (**Fig. 8C**).

In order to understand the role of SphK-1 in TNF-α secretion in macrophages more precisely, we stimulated WT and Sphk-1++ macrophages with bacterial LPS to stimulate TNF-α secretion. Consistent with NO, DHS treatment inhibited LPS induced TNF-α secretion significantly in WT macrophages, but contrary to NO generation, we found negative regulation of SphK-1 overexpression on TNF-α secretion in LPS stimulated SphK-1++ macrophages (**Fig. 8D**). Like before, S1P treatment delayed LPS induced secretion of TNF-α also in SphK-1++ macrophages significantly in comparison to WT macrophages at 9 h (**Fig. 8D**), which was restored at 24 h (**Fig. 8E**). Co-stimulation of infected macrophages with LPS enhanced TNF-α secretion synergistically in WT macrophages (**Fig. 8F**), whereas SphK-1++ macrophages remained resistant to such co-stimulation and TNF secretion in their culture supernatant remained lower in comparison to WT macrophages. These observations revealed the dual fate of SphK-1 modulation upon TNF-α secretion from macrophages and were found to be consistent with other recent findings [59], [60].

### M. smegmatis infection induces S1P production

**Figure 9:**
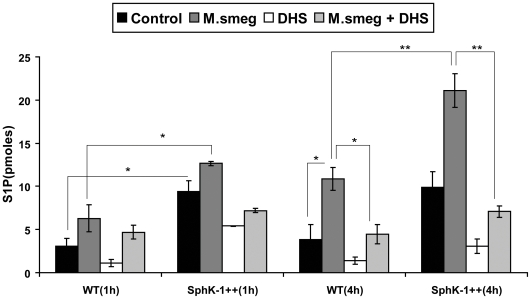
*M. smegmatis* infection enhances S1P production in macrophages. Both WT and SphK-1++ were infected with *M. smegmatis* with and without DHS and S1P titres were quantified. Data are represented as a mean of ρmoles of S1P ± SEM produced in each group from two independent experiments. ** Indicates p≤0.01; * indicates p≤0.05.

SphK activity induces the formation of S1P, which is known for its antimycobacterial characteristics [61]. Therefore, we quantified S1P titers among WT and SphK-1++ infected macrophages at 1 h and 4 h post infection. *M. smegmatis* infection induced S1P production significantly in both WT and SphK-1++ macrophages (**Fig. 9**), suggesting an increase of SphK activity in these macrophages. This observation was found to be in agreement with recent finding [18]. As expected, the S1P titre was found to be elevated in the infected SphK-1++ macrophages. The S1P titers in both control and infected macrophages were inhibited significantly by treatment with DHS (**Fig. 9**), reflecting a decrease in SphK activity in these macrophages. This observation also validated the inhibition of sphingosine kinase activity by DHS in these macrophages. In WT and Sphk-1++ infected macrophages, the S1P levels expectedly remained higher in Sphk-1++ macrophages even after their treatment with DHS. This indicated the significance of the elevation of S1P levels upon activation of macrophages, since S1P is known to contribute to the survival of macrophages [62]–[64].

## Discussion

Mycobacterial infections represent the third major cause of worldwide annual mortalities [65]–[68] and have raised serious concerns about the development of effective therapies for controlling infection. Recent reports have shown the involvement of SphK-1 during mycobacterial infections in macrophages [18], but the key question of whether SphK-1 can actually control mycobacterial infections remains unanswered.

We observed the increased sensitivity to infection of macrophages deprived of SphK-1 (both DHS treated and Si-Sphk-1 knockdown), demonstrating a novel antimycobacterial role of SphK-1 in infected macrophages. Sphk-1 is known to induce a pro-inflammatory environment [69], [70] and phagolysosome maturation [71] in macrophages. Reduced expression of major phagocytic and antimycobacterial proteins in Sphk-1 knockdown macrophages clearly indicated their poor innate immune defense against mycobacteria, which rendered these macrophages sensitive to infection, Reduced activation of the RelA subunit (pp65) of NF-κB in Sphk-1 knockdown and infected WT macrophages further suggested a possible regulation of Sphk-1 of both NF-κB itself and NF-κB mediated signaling (iNOs proteins, NO generation, TNF secretion and LAMP-2), which are important and involved in mycobacterial killing in macrophages [21]. Increased expression of LAMP-2, iNOs and p-p38 in SphK-1++ macrophages in response to *M. smegmatis* infection suggested increased antimycobacterial potential of Sphk-1++ macrophages, providing the most probable evidence of increased resistance of these macrophages to infection. As predicted, control sphingolipids did not affect macrophage response either to *M. smegmatis* infection or LPS/TNF stimulation. This was due to the fact that the same lipid (including other related control lipids) did not alter translocation of CD69 (β-actin receptor) and Nf-kB activation in macrophages in response to *M. smegmatis* infection [72], which are required for the bacterial killing and stimulation of macrophages.

The antimycobacterial role of NO has been documented in macrophages [32]–[34]. Reduced generation of NO in macrophages deprived of SphK-1 demonstrated their poor activation since NO is a well-known and potent marker of activated M-1 macrophages [73], [74]. Transcriptional regulation of iNOS is very complex and more than 15 different consensus binding sites for transcription factors have been identified in the promoter region of the *iNOS* gene. One of the major factors regulating *iNOS* transcription is NF-κB. Reduced activation of NF-κB indeed provided a potential reason for reduced NO titre in Sphk-1 knockdown macrophages. Reduction of *M. smegmatis* infection or LPS induced NO generation by DHS and its complementation by SphK-1 overexpression clearly indicated that SphK-1 is indispensable for the generation of NO in macrophages. Increased NO titre and expression of iNOs proteins in SphK-1++ macrophages demonstrated moreover their M-1^bright^ phenotype, which are more competent and bear efficient antibacterial defenses [31].

Reduced bacterial uptake, growth and enhanced NO generation in SB 203580 treated and infected macrophages over infected macrophages could possibly be due to the regulatory role of p38MAPK over mycobacterial growth [53], [56], negative regulation of expression of the early endosomal marker (EEA-1) [75] and generation of NO [55]. Increased uptake and enhanced bacterial growth in infected and SB treated macrophages by DHS revealed a novel regulation of infection by SphK-1 over p38 in infected macrophages. The similar regulation of SphK-1 over p38 was also shown by the inhibition of *M. smegmatis* and SB 203580 induced titre of NO by DHS. These results clearly depicted the novel regulation of SphK-1 over p38 MAPK, which is quite important for host innate immunity [76], [77].

DHS mediated inhibition of TNF-α secretion in infected WT macrophages rendered an anti-inflammatory environment [59] and provided the second major reason for the sensitization of Sphk-1 inhibited macrophages to infection. Reduced TNF-α levels in SphK-1++ infected macrophages also reflected the anti-inflammatory effect of SphK-1 overexpression in the macrophages as observed earlier.[78] The dual regulation of SphK-1 on TNF-α secretion was explicitly demonstrated by the inhibition of either LPS or *M. smegmatis* induced TNF-α secretion in both WT and SphK-1++ RAW macrophages by both S1P and DHS and was found to be in agreement with other recent finding [60].

Despite the low TNF-α titre in the infected SphK-1++ macrophages, their increased resistance to infection was found to be due to the enhanced expression of LAMP-2, iNOs, p-p38 and the elevated S1P titre, which all together conferred optimum defenses in these macrophages against *M. smegmatis* in a TNF independent manner. Due to the antimycobacterial nature of S1P [61], the elevated S1P titre in infected SphK-1++ macrophages over infected WT macrophages provided yet more compelling evidence for their enhanced antimycobacterial potential. These findings thus demonstrate that SphK-1 is capable of controlling mycobacterial infection in macrophages.

## Materials and Methods

### Reagents and antibodies

DMEM with high glucose, Lipopolysaccharide (LPS) from *Salmonella typhimurium*, Gentamycin, Geniticin (G418), NaNO_2, D-_Sphingosine, Sphingosine 1 phosphate, ***D, L erythro dihydrosphingosine*** and the SphK-1 inhibitor ***D, L-threo-Dihydrosphingosine*** **(DHS)** were purchased from Sigma (Taufkirchen, Germany). The TNF-α OptEIA™ ELISA kit was purchased from BD Pharmingen (Heidelberg, Germany). SB 203580 (p38 inhibitor) was purchased from Cal-Biochem (Darmstadt, Germany). 7H9 medium was purchased from Difco (Karlsruhe, Germany). Sulphanilamide and N-(naphthyl) ethylene-diaminedihydrochloride were purchased from E-Merck (Darmstadt, Germany). Rabbit anti-mouse Sphingosine kinase-1 polyclonal antibody was purchased from Cayman chemicals (Hamburg, Germany). Mouse monoclonal iNOs antibody was purchased from BD transductions (Heidelberg, Germany). Rabbit anti-mouse pp38 antibody was purchased from Santa Cruz (Heidelberg, Germany). Rat anti-mouse LAMP-2 was purchased from Iowa Hybridoma Bank. Rabbit anti-mouse pp65 (RelA) antibody was purchased from Cell signaling. Goat anti-rabbit Alexa flour 488, Goat anti-rat-Cy3 and Goat anti-mouse-Cy3 conjugated antibodies were purchased from Di-nova (Koenigswinter, Germany). Mouse Sphk-1 specific siRNA duplex was purchased from Santa Cruz Biotechnology. Control siRNA duplex was purchased from Qiagen.

### Sphk-1 knockout mice

Sphk-1 KO mice (C57Bl6/j background) were established, housed and bred at the animal housing facility at SUNY Downstate and VA Medical Center, NewYork, in a pathogen-free environment. These mice were originally developed by Richard Proia, NIDDK Bethesda, USA [79] and further characterized by Wadgaonkar et al., SDVAMC, New York, USA, in their lung injury model [80].

### Cell culture

RAW 264.7 macrophages were maintained in Dulbecco Modified Eagle Medium supplemented with 4.5 g/l of glucose, 10% FCS, penicillin and streptomycin (1%). The SphK-1++ RAW 264.7 macrophages were maintained in G418 containing DMEM medium. For mycobacterial infection, (0.5×10^6^) macrophages were cultured overnight in antibiotic free medium and infected the next day at the density of 1×10^6^. Unless otherwise mentioned, all experiments were conducted in medium containing DHS. Mouse peritoneal macrophages were isolated by a standard procedure from WT C57Bl6/_j_ and SphK-1 KO mice. To isolate peritoneal macrophages, the mice were injected 4% thioglycolate medium intraperitoneally and peritoneal lavage was collected 72 h post injection. Next, the CD11b+ macrophages in peritoneal lavage were purified by MACS based positive selection method using CD11b+ magnetic micro beads (Miltenyi Biotech). 1×10^6^ CD11b+ peritoneal macrophages per well were cultured in a final volume of 200 µl RPMI 1640 medium in flat-bottom 96-well polystyrene microtitre plates (Corning) and incubated at 37°C and 5% CO_2_ overnight for adherence to plastic surface. Next day the adherent macrophages were used for various experiments.

### Sphk-1 overexpression

SphK-1 was overexpressed in RAW macrophages. For this purpose, SphK-1 gene was sub-cloned into pEGFP-N1 vector. The primers which were used for cloning had the following sequences: 5′-CCCGAATTCATGGAACCAGAATGCCCTCGA. 3′-primer: GCGTCTAGATTATGGTTCTTCTGGAGTTGG. The macrophages were transfected with SK-1-GFP using EcoR1/Xba1 bearing neomycin resistant cassette. The cells were positively selected Geniticin G418 (600 µg/ml) containing medium. The GFP expression was validated by FACS, and SphK-1 overexpression was confirmed by western blot, immunofluorescence staining and competitive S1P titre (**Fig. 2A**).

### SiRNA knockdown of Sphk-1

Sphk-1 in RAW macrophages was knocked down using commercially available Sphk-1 siRNA duplex from Santa Cruz (Sc-45446) with the following sequences: 5′UTR CUGGUGUUAUGCAUCUGUU, GCAAGCAUAUGGAACUUGA and CCUUCCAGUUAGAGUAACA, together with scrambled siRNA duplex (Qiagen). The cells were transfected with these duplexes using siRNA transfection reagent (Santa Cruz Biotech) as recommended by the manufacturer in serum and antibiotic-free medium. The knockdown was confirmed 24 h post transfection by western blot (**Fig. 1F**).

### Cell survival and metabolic activity

The survival of macrophages under experiments was evaluated by the MTT dye reduction method. After each incubation time the cells were incubated with yellow MTT dye [3-(4, 5-dimethylthiazol-2-yl)-2, diphenyl-tetrazolium bromide (Sigma, Taufkirchen, Germany) (20 µl/well of 5 mg/ml) for 1 h at 37°C in a CO_2_ incubator. The Formazone crystals (reduction product of dye by mitochondrial reductase) were dissolved in DMSO and ethanol and OD was measured at 570 nm in a spectrometer Spectra max 250 (Molecular Devices, Munich, Germany). Similarly, the metabolic activity of macrophages was determined by WST-1 assays (Roche Diagnostics, Mannheim, Germany). 20 µl of reagent was added to a 0.2 ml volume of cell culture and incubated for 1 h and the absorbance was measured at 440 nm in a spectrometer, Spectra max 250 (Molecular Devices, Munich, Germany).

### *M. smegmatis* infection

Log phase cultures of *M. smegmatis* were used for infecting macrophages. The bacterial cultures were maintained in 7H9 medium supplemented with 0.2% glycerol. Before infecting macrophages, the bacterial clumps were removed by repeatedly washing and sonicating bacterial suspension mildly for 15 min at room temperature in a mild water sonicater bath as described previously [23], [24], [81]. Before infecting cells with these bacteria, the viability of single bacterial cell suspension was checked under a light microscope. WT, SphK-1 KD (knockdown) and SphK-1++ macrophages were infected with *M*. *smegmatis* in antibiotic free medium for 1 h. After 1 h infection period, the medium was replaced with medium containing Gentamycin (10 µg/ml) to get rid of extracellular bacteria. The cells were incubated further for various time intervals. After each incubation interval, the cells were washed twice with PBS to remove extracellular bacterial and cellular debris and lysed in distilled H_2_O. The infection was quantified by a CFU (colony forming unit) based method as previously described [16], [23]. For this purpose, serially diluted cell lysates were platted on LB agar plates in triplicate. The bacterial colonies were counted after 2–3 days and infection was documented as colonies (CFU) per milliliter.

### Western blotting

The cells were lysed in 50 mM Tris-HCl (pH 7.4), 150 mM NaCl, 2 mM EDTA, 1% Nonidet P-40 and complete protease inhibitor cocktail (Roche) and sonicated with 35 pulses of 50 milliseconds. Protein concentrations in samples were determined by the Bradford method (Bio-Rad, Munich, Germany). 20 µg of proteins per sample were separated on gradient gel (Nu-PAGE) and blotted on PVDF membrane by wet electro-blotting method. The membranes were blocked with 5% non-fat dry milk in TBS-T; pH 7.5 (20 mM Tris base, 137 mM NaCl, and 0.1% T-ween20) and incubated with primary antibody overnight at 4°C. This was followed by incubation with the HRP conjugated secondary antibody. Blots were developed by ECL reagent (Amersham, Life Sciences Freiburg, Germany) and normalized against actin.

### Immunofluorescence staining

The expression of SphK-1, iNOs, pp38 and LAMP-2 markers in macrophages were visualized by immunofluorescence staining. For immunostaining, both WT and SphK-1++ macrophages were infected on cover slips. The cells were fixed with 4% PFA for 15 min and washed once with 1×TBS. The cells were then permeabilized by TBST-Triton ×100 (0.1%) for 5 min and blocked with TBS + 1% BSA+ 1% fish gelatin for 30 min. After three washes with TBS, cells were incubated with primary antibodies for 1 h at RT. This was followed by incubation of cells with secondary antibody for 1 h in the dark. The cells were counter stained with DAPI to stain nucleus. The cells were mounted and analyzed under fluorescent microscope (Axivort, Carl Zeiss) under 100× magnification.

### NO quantification

Supernatants from macrophages under observation were used to determine NO as NO_2_ by standard Griess reagent assay. Equal volumes of the culture supernatants and Griess reagent (1%sulphanilamide/0.1%N-(naphthyl) ethylene-diaminedihydrochloride prepared in 5% o-phosphoric acid) were mixed and absorbance was measured at 550 nm by Spectra max spectrometer (Molecular Devices ORT). The NO titres in samples were quantified against a NaNO_2_ standard curve generated using software provided with the Spectra max spectrometer (Molecular Devices).

### TNF-α measurement

TNF-α titre in culture supernatants of macrophages was quantified by using the standard OptEIA™ ELISA kit (BD Pharmingen, USA) as per the instruction manual. The TNF titers in samples were calculated by SPF software using a cytokine standard generated standard curve.

### S1P quantification

S1P was measured as previously described [82]. Briefly, the cell monolayers were scraped and washed once with ice cold PBS. The cell pallets were dissolved in methanol containing concentrated HCl. The lipids were extracted by a modified two-step extraction method by addition of chloroform and 1N NaCl. For alkalization a 3N NaOH solution was added. After centrifugation (300 g, 5 min), the alkaline aqueous phase containing S1P was transferred into siliconized glass tubes. The organic phase was re-extracted with methanol, 1N NaCl and 3N NaOH. The aqueous phase was acidified with concentrated HCl and extracted twice with chloroform. The combined organic phase was evaporated using a vacuum system (Savant, Bethesda, Md., USA). The dried lipids were then resolved in methanol/0.07 M K_2_HPO_4_ (pH-8.2) by rigorous vortexing and sonicating on ice for 5 min. A derivatization mixture of o-phthaldialdehyde, mercaptoethanol, ethanol and boric acid solution (pH-10.5) was prepared and added to the lipid fractions (resolved in methanol/0.07 M K_2_HPO_4_) for 15 min at room temperature. The derivatives were analyzed using the Merck Hitachi LaChrom HPLC system (Merck Hitachi, Darmstadt, Germany). Fluorescence was measured at 340 nm after separation on RP-18 Kromasil column (Chromatographie Service, Langerwehe, Germany). The flow rate was adjusted to 1.3 ml/min and a gradient program was prepared with methanol and 0.07 M K_2_PO_4_ as solvents and applied (Table 1). Dihydro S1P was used as an internal control and S1P was quantified in each sample by using the Merck system manager software.

**Table 1:** Gradient program for S1P and dihydro-S1P separation utilized for the HPLC analysis of S1P.

Time (min)	0.07 M K_2_PO_4_ (%)	Methanol (%)
0	24	76
10	24	76
30	16	84
40	8	92
46	0	100
56	0	100
58	24	76

### Statistical analysis

The averages and standard errors of the mean as well as the student's t-tests have been calculated; significance is indicated with ** p<0.01 and * p<0.05.

## Supporting Information

Figure S1 Killing of M. smegmatis by RAW macrophages. 1×106 RAW macrophages were infected with M. smegmatis (MOI-1 and 5) and mycobacterial killing was monitored up to 4 h post infection. Data are represented as mean of CFU ± SEM from three independent experiments. ** Indicate p<0.01; * indicate p<0.05.
(0.92 MB TIF)

Figure S2 Effect of DHS on the survival and stimulation of macrophages (A) Both WT and Sphk-1++ macrophages were treated with varying doses of DHS and their survival was monitored at different time intervals by MTT dye reduction method as described. The OD was taken at 570 nm by a spectrophotometer. (B) The metabolic activity of the macrophages under section (A) was measured by WST-1 dye reduction method as described. The OD was measured at 440 nm by spectrophotometer. Data are represented as mean of OD ± SEM from three independent experiments.
(0.81 MB TIF)

Figure S3 Effect of control lipids on the killing of M. smegmatis by RAW macrophages Macrophages were treated with control sphingosine derivatives D-sphingosine (A) or D-erythro dihydrosphingosine (B) and infected with M. smegmatis and mycobacterial killing was monitored up to 24 h post infection. Data are represented as mean of CFU ± SEM from three independent experiments. (C) The macrophages were infected in the presence of either sphingosine or D, L erythro- dihydrosphingosine (no SK inhibitory activity) and NO was compared among different groups at indicated time intervals. Data are represented as mean of µM ± SEM from three independent experiments. The dotted line in the figure represents and cuts-off the constitutive NO titre in macrophages. The values above this line represent the actual titre of NO induced by various treatments.
(0.59 MB TIF)

Figure S4 S1P regulates M. smegmatis infection induced NO generation in macrophages. (A) Both WT and SphK-1++ macrophages were infected with M. smegmatis with and without S1P/DHS and NO was quantified at indicated time intervals. (B) Both WT and SphK-1++ macrophages were stimulated with various stimuli (LPS/TNF/IFN/SNP) with and without DHS for indicated time intervals and NO was quantified in their culture supernatants at 24 h post treatment. (C) Both WT and SphK-1++ macrophages were stimulated with (LPS/TNF/IFN/S1P) with and without iNOs specific modulators (SNP/LNMA) and NO was quantified at 24 h post stimulation. (D) SphK-1 overexpression enhances the expression of iNOs proteins in macrophages. WT and Sphk-1++ macrophages were stimulated with various stimuli (LPS/TNF-α/IFN-γ) with and without S1P/DHS: The expression of iNOs proteins was analyzed at 24 h. Shown here is the representative blot from two independent experiments. Data are represented asµM ± SEM from three independent experiments. The dotted line in the figure represents and cuts-off the constitutive NO titre in macrophages. The values above this line represent the actual titre of NO being induced by various treatments.
(2.24 MB TIF)

Figure S5 Effect of control lipids on LPS and/or TNF-α induced NO in macrophages. RAW macrophages were stimulated with either LPS (A) or TNF-α (B) with and without d-sphingosine and d, l-erythro dihydrophingosine (DHS-related sphingosine-derivative without SK inhibitory activity) for indicated time intervals. The NO was quantified in their culture supernatants. The dotted line in the figure represents and cuts-off the constitutive NO titre in macrophages. The values above this line represent the actual titre of NO being induced by various treatments. Data are represented as µM ± SEM from two independent experiments.
(0.98 MB TIF)
