# Supplementary figures and images for: Sphingosine Kinase-1 (SphK-1) Regulates Mycobacterium smegmatis Infection in Macrophages
Source: PLoS One. 2010 May 17;5(5):e10657. doi: 10.1371/journal.pone.0010657 (PMC2871783; doi:10.1371/journal.pone.0010657)

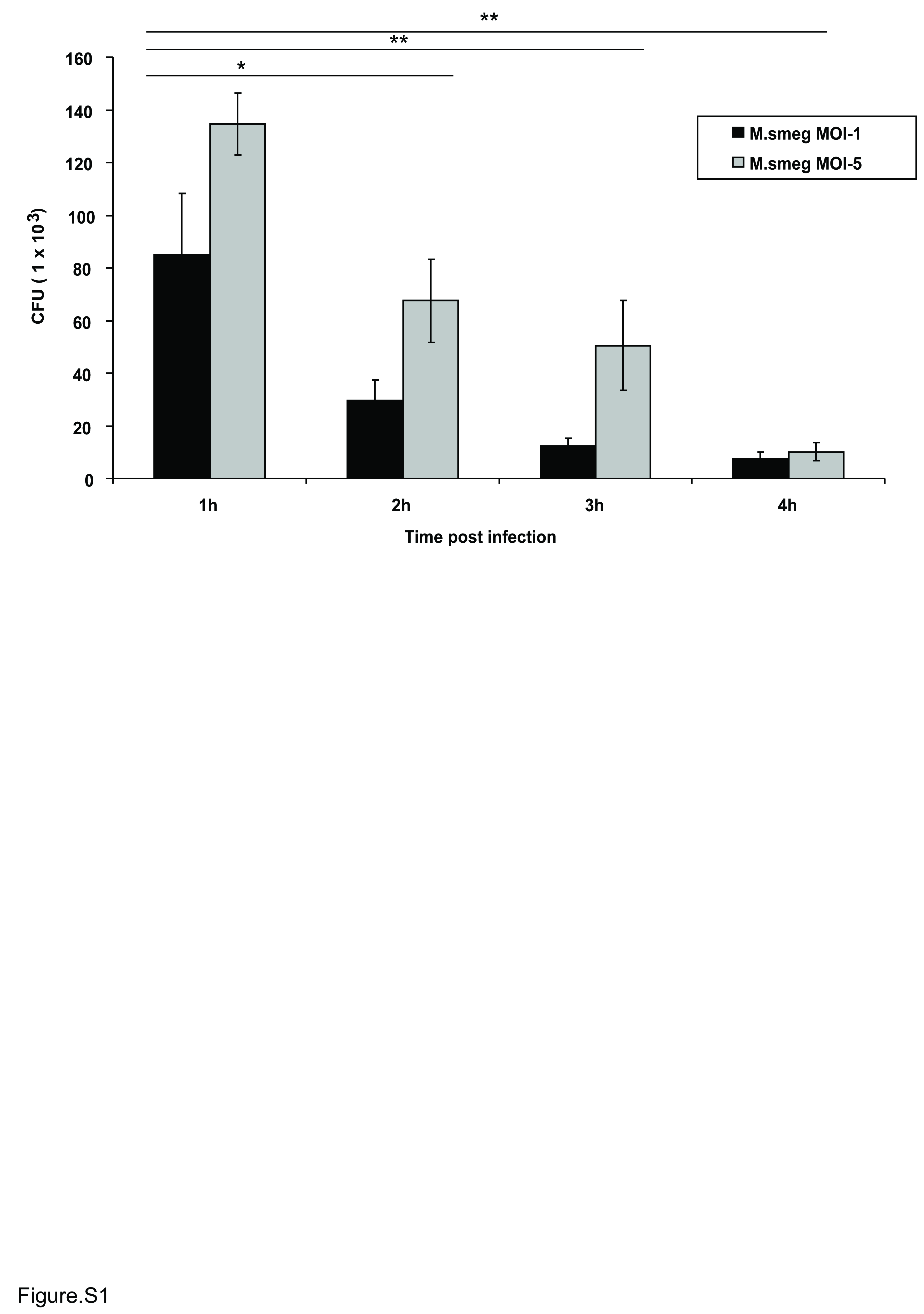

Supplement: Figure S1 — Killing of M. smegmatis by RAW macrophages. 1×10^6^ RAW macrophages were infected with M. smegmatis (MOI-1 and 5) and mycobacterial killing was monitored up to 4 h post infection. Data are represented as mean of CFU ± SEM from three independent experiments. ** Indicate p<0.01; * indicate p<0.05. (0.92 MB TIF) [file pone.0010657.s001.tif]

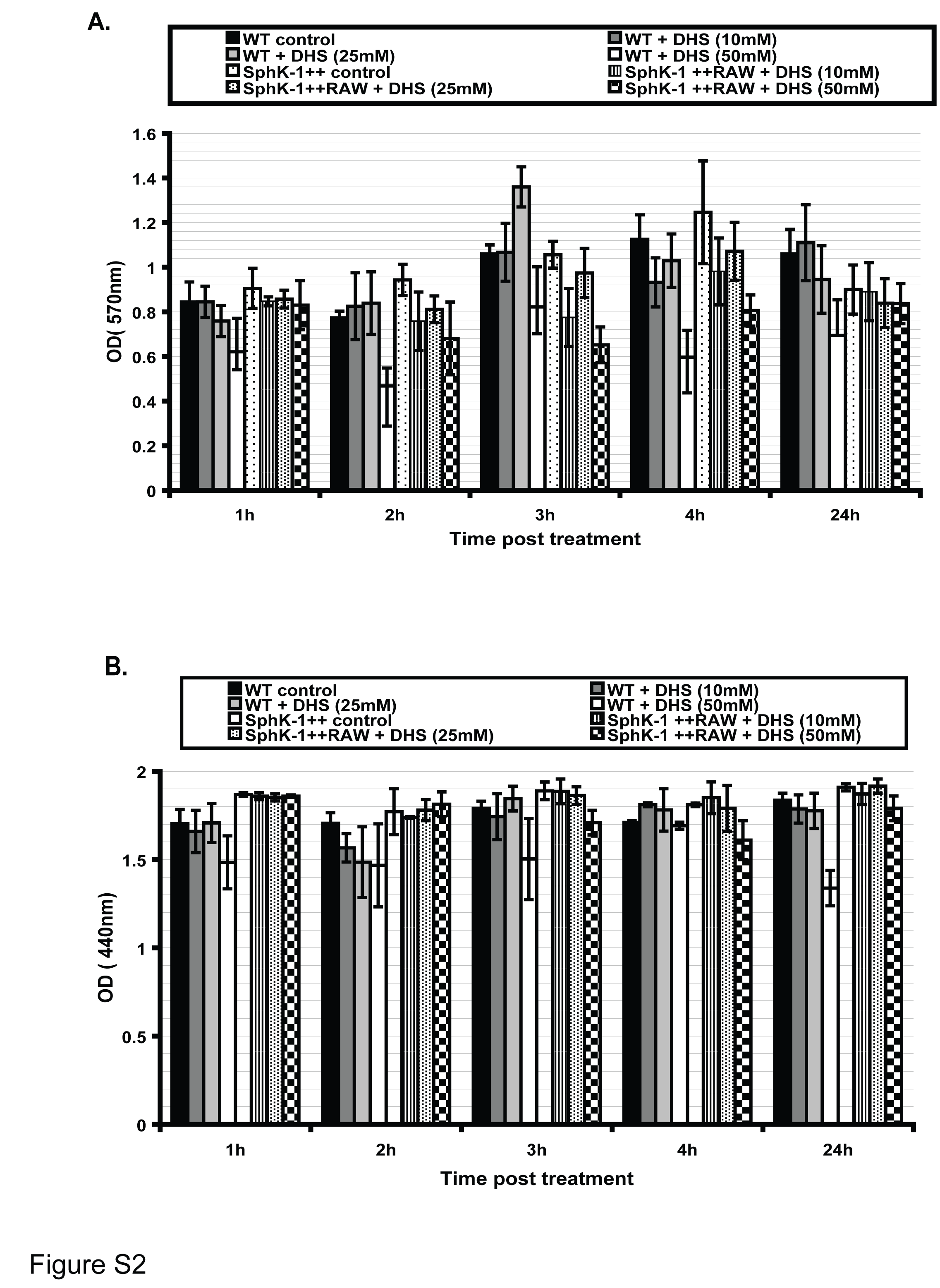

Supplement: Figure S2 — Effect of DHS on the survival and stimulation of macrophages (A) Both WT and Sphk-1++ macrophages were treated with varying doses of DHS and their survival was monitored at different time intervals by MTT dye reduction method as described. The OD was taken at 570 nm by a spectrophotometer. (B) The metabolic activity of the macrophages under section (A) was measured by WST-1 dye reduction method as described. The OD was measured at 440 nm by spectrophotometer. Data are represented as mean of OD ± SEM from three independent experiments. (0.81 MB TIF) [file pone.0010657.s002.tif]

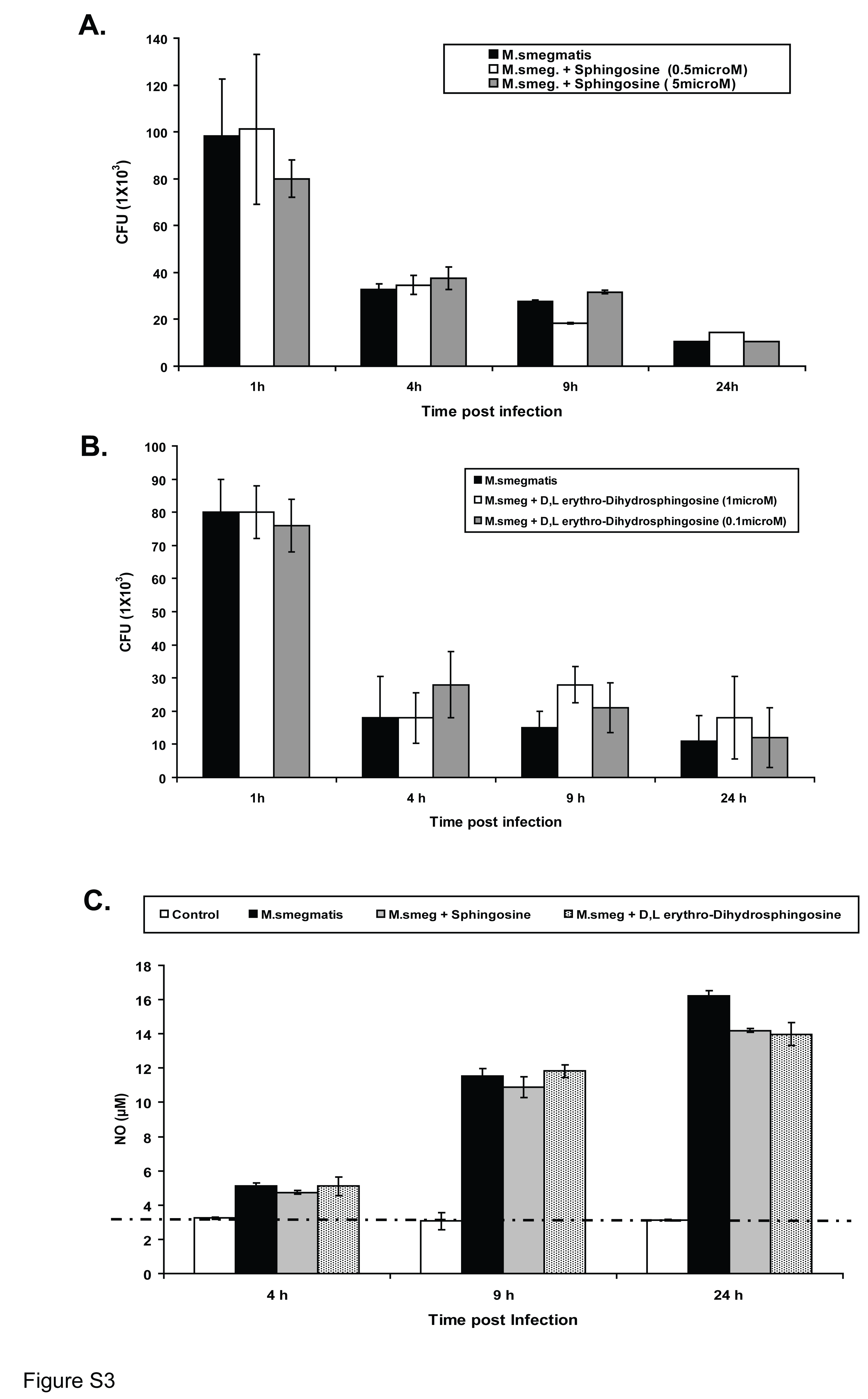

Supplement: Figure S3 — Effect of control lipids on the killing of M. smegmatis by RAW macrophages Macrophages were treated with control sphingosine derivatives D-sphingosine (A) or D-erythro dihydrosphingosine (B) and infected with M. smegmatis and mycobacterial killing was monitored up to 24 h post infection. Data are represented as mean of CFU ± SEM from three independent experiments. (C) The macrophages were infected in the presence of either sphingosine or D, L erythro- dihydrosphingosine (no SK inhibitory activity) and NO was compared among different groups at indicated time intervals. Data are represented as mean of µM ± SEM from three independent experiments. The dotted line in the figure represents and cuts-off the constitutive NO titre in macrophages. The values above this line represent the actual titre of NO induced by various treatments. (0.59 MB TIF) [file pone.0010657.s003.tif]

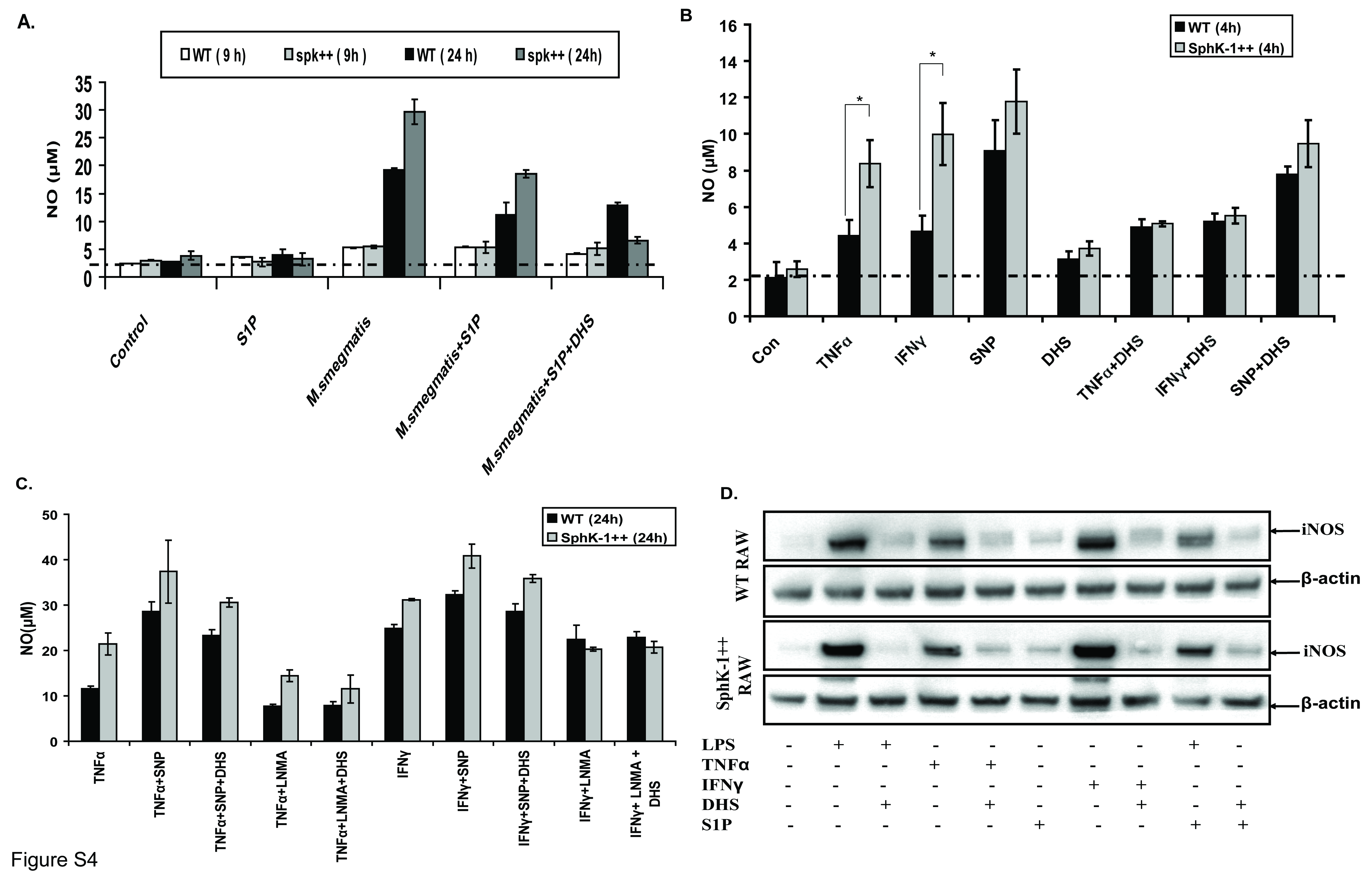

Supplement: Figure S4 — S1P regulates M. smegmatis infection induced NO generation in macrophages. (A) Both WT and SphK-1++ macrophages were infected with M. smegmatis with and without S1P/DHS and NO was quantified at indicated time intervals. (B) Both WT and SphK-1++ macrophages were stimulated with various stimuli (LPS/TNF/IFN/SNP) with and without DHS for indicated time intervals and NO was quantified in their culture supernatants at 24 h post treatment. (C) Both WT and SphK-1++ macrophages were stimulated with (LPS/TNF/IFN/S1P) with and without iNOs specific modulators (SNP/LNMA) and NO was quantified at 24 h post stimulation. (D) SphK-1 overexpression enhances the expression of iNOs proteins in macrophages. WT and Sphk-1++ macrophages were stimulated with various stimuli (LPS/TNF-α/IFN-γ) with and without S1P/DHS: The expression of iNOs proteins was analyzed at 24 h. Shown here is the representative blot from two independent experiments. Data are represented asµM ± SEM from three independent experiments. The dotted line in the figure represents and cuts-off the constitutive NO titre in macrophages. The values above this line represent the actual titre of NO being induced by various treatments. (2.24 MB TIF) [file pone.0010657.s004.tif]

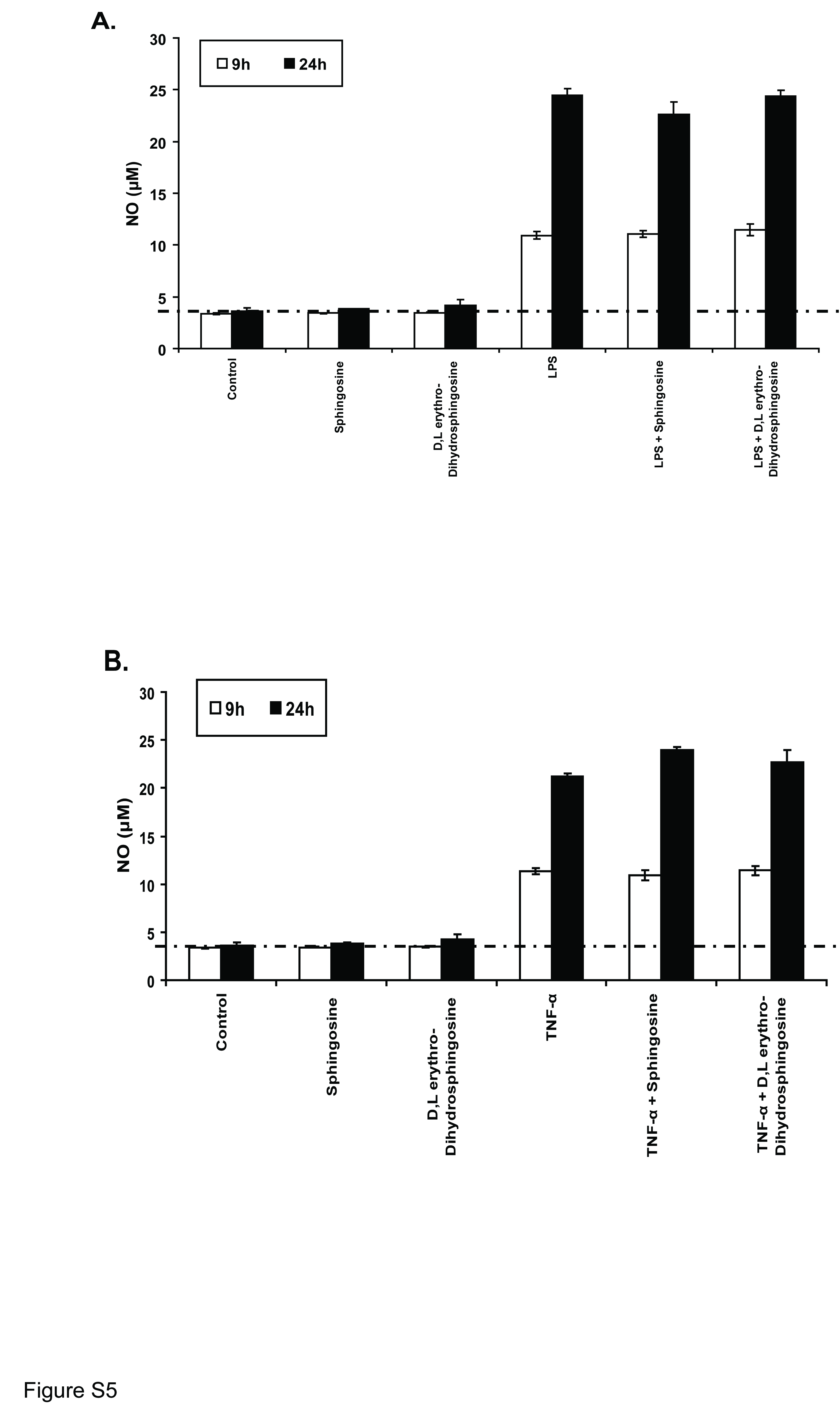

Supplement: Figure S5 — Effect of control lipids on LPS and/or TNF-α induced NO in macrophages. RAW macrophages were stimulated with either LPS (A) or TNF-α (B) with and without d-sphingosine and d, l-erythro dihydrophingosine (DHS-related sphingosine-derivative without SK inhibitory activity) for indicated time intervals. The NO was quantified in their culture supernatants. The dotted line in the figure represents and cuts-off the constitutive NO titre in macrophages. The values above this line represent the actual titre of NO being induced by various treatments. Data are represented as µM ± SEM from two independent experiments. (0.98 MB TIF) [file pone.0010657.s005.tif]
